# Comorbidity Burden in Adults With Autism Spectrum Disorders and Intellectual Disabilities—A Report From the EFAAR (Frailty Assessment in Ageing Adults With Autism Spectrum and Intellectual Disabilities) Study

**DOI:** 10.3389/fpsyt.2019.00617

**Published:** 2019-09-19

**Authors:** Stéphanie Miot, Tasnime Akbaraly, Cecile Michelon, Sylvie Couderc, Sophie Crepiat, Julie Loubersac, Marie-Christine Picot, Éric Pernon, Véronique Gonnier, Claude Jeandel, Hubert Blain, Amaria Baghdadli

**Affiliations:** ^1^CESP, INSERM U1178, Centre de recherche en Epidemiologie et Santé des Populations, Paris, France; ^2^Autism Resources Centre of Languedoc-Roussillon, University Hospital of Montpellier, CHRU de Montpellier, Univ. Montpellier, Montpellier, France; ^3^Gerontology Centre, Antonin Balmès, University Hospital of Montpellier, CHRU de Montpellier, Univ. Montpellier, Montpellier, France; ^4^MMDN, Univ. Montpellier, EPHE, INSERM, U1198, Montpellier, France; ^5^Department of Epidemiology and Public Health, University College London, London, United Kingdom; ^6^Biostatistic Department, University Hospital of Montpellier, CHRU de Montpellier, Univ. Montpellier, Montpellier, France

**Keywords:** autism spectrum disorder, intellectual disability, ageing, comorbidity burden, CIRS

## Abstract

**Background:** Autism spectrum disorder (ASD) is an early-onset and lifelong neurodevelopmental condition frequently associated with intellectual disability (ID). Although emerging studies suggest that ASD is associated with premature ageing and various medical comorbidities, as described for ID, data are scarce.

**Objectives:** To determine the comorbidity burden and its association with distinct clinical presentation in terms of ASD severity, adaptive skills, level of autonomy, and drug exposure in a well-phenotyped sample of individuals with ASD-ID—the EFAAR (Frailty Assessment in Ageing Adults with Autism Spectrum and Intellectual Disabilities) cohort.

**Methods:** A total of 63 adults with ASD-ID, with a mean age of 42.9 ± 15.1 years, were recruited from 2015 to 2017 from nine specialized institutions. They underwent detailed clinical examinations, including screening for comorbidities, ASD severity [Childhood Autism Rating Scale (CARS)], adaptive functioning [Vineland Adaptive Behavior Scale II (VABS-II)], autonomy [activities of daily living (ADLs)], and drug use [polypharmacy and the Drug Burden Index (DBI)]. The comorbidity burden was evaluated using the Cumulative Illness Rating Scale (CIRS-G) and its sub-scores [the severity index (CIRS-SI) and severe comorbidity (CIRS-SC)].

**Results:** We found a large range of comorbidities, including gastrointestinal disorders and mental and neurological diseases. Overall, 25% of our ASD-ID sample had chronic kidney disease with the associated increased cardiovascular risk factors. The comorbidity burden was high (mean CIRS-G total score of 10.6 ± 4.8), comparable with that observed among patients older than those in our population hospitalized in geriatric departments. Furthermore, the comorbidity burden positively correlated with age, decreased autonomy, and polypharmacy.

**Conclusion:** The severity of the comorbidity burden associated with premature ageing in adults with ASD and ID highlight their crucial need of personalized medical care.

## Introduction

Ageing is a dynamic process, resulting in decreased physiological reserves that can lead to impaired adaptive capacities in elderly individuals. In the general population, ageing results in increased multimorbidity (defined as two or more chronic conditions) (1), leading to disability (2), polypharmacy (defined as five or more medications per day) (3), and mortality (4).

Autism spectrum disorder (ASD) is a neurodevelopmental disorder characterized by social and communication impairment associated with repetitive and restrictive behaviors (5). One individual in 68 has an ASD in the United States (6) and one in 100 in France (7), making it a relatively common condition (8). Its clinical presentation is heterogeneous, and psychiatric and somatic comorbidities are both variable and frequent (9).

Aside from ASD patients having a higher mortality rate than that of the general population, little is known about ageing in ASD (9). Several studies have hypothesized a pathological ageing trajectory in ASD (10, 11), related to a high rate of comorbidities, particularly feeding (12, 13) and gastrointestinal disorders (14, 15), which have been reported in almost 90% of cases. Seizure disorders (16), immune dysregulation (17), and cardiovascular diseases (18) are also common and reported in one third of individuals.

In addition, intellectual disability (ID), found in 32% of ASD individuals (9), is commonly associated with a large range of medical comorbidities, such as nutritional deficiencies, cardiovascular diseases (18), polypharmacy (19), and multi-morbidity (20), and may contribute to the increased risk of premature ageing of ASD patients (11, 21, 22).

We hypothesized that the cumulative weight of comorbidity associated with ASD and ID may lead to premature ageing of adult patients with ASD and comorbid ID (ASD-ID). However, there have been few observational studies to investigate the impact of comorbidities on ageing trajectories in adults with ASD. Here, we aimed to determine the comorbidity burden in a well-phenotyped cohort of adults with ASD-ID, the EFAAR (Frailty Assessment in Ageing adults with Autism Spectrum and Intellectual Disabilities) cohort, using the Cumulative Illness Rating Scale (CIRS-G) and its sub-scores [the severity index (CIRS-SI) and severe comorbidity (CIRS-SC)]. We explored the predictive factors of such a comorbidity burden in terms of age, ASD severity, adaptive functioning, autonomy, and drug use. Secondary objectives were to better characterize the medical comorbidities associated with ASD-ID during adulthood and the pre-elderly period and determine those comorbidities that are more frequently associated with each clinical feature.

## Materials and Methods

### Study Design and Population

The EFAAR study is an ongoing prospective multicentric study. Participants were recruited from nine medico-social institutions in the south of France between 2015 and 2018. These institutions are the place of both residence and care of participants. Participants with a diagnosis of ASD [according to the *Diagnostic and Statistical manual of Mental Disorders* (DSM-5) criteria] and an ID [established according to the American Psychiatric Association (APA, 2013)] were invited to participate in the EFAAR study. Inclusion criteria included being over the age of 20 years and being institutionalized in a medico-social institution of Languedoc-Roussillon (South of France). The exclusion criterion was having Down syndrome, known to be a cause of premature ageing (23). Among the 65 participants (recruited in nine centers), two aged 65 years were excluded (one declined and one dropped out after moving away from Languedoc Roussillon). In total, the EFAAR cohort included 63 participants who underwent a thorough clinical examination at baseline focused on frailty assessment. They will be followed up over 5 years, during which time certain health events will be recorded annually through phone interviews with the health workers (falls, hospitalizations, and death). The present study is based on baseline examination data.

### Baseline Examinations

Baseline examinations were carried out within the medico-social institution of the participant to reduce anxiety due to the assessment and evaluate each patient during a stable phase of their disease.

ASD severity was assessed using the Childhood Autism Rating Scale (CARS) (24), a standardized scale that evaluates the intensity of autism symptoms across 15 domains, each scored from 1 to 4. The total score is the sum of each of the 15 sub-scores (range 15–60, with a higher score indicating higher severity). This evaluation was completed by three of the authors (SM, SC, or SC). The three investigators reached a consensus to determine the CARS total score.

The intellectual quotient (IQ) was assessed using the Raven Progressive Matrices. However, none of the participants could understand the test instructions or requirements. A developmental quotient (DQ) was calculated to confirm the ID, according to Stern’s formula (25): developmental age (defined based on the daily life sub-score of the VABS-II)/chronological age * 100.

Adaptive functioning was assessed by the Vineland Adaptive Behavior Scale II (VABS-II) (26), a semi-structured interview conducted with the health worker of the participant. Three sub-scores (communication, daily life, and social skills) (Vineland II, 2004) were calculated, with a higher score indicating a less severe impairment of adaptive functioning (27).

Autonomy was assessed using the Katz index of independence for six activities of daily living (ADLs), which included bathing, dressing, toileting, transferring, continence, and feeding (28). A score of 1 (if the patient needs no assistance for the specific ADL), 0.5 (if the patient needs supervision, direction, or assistance), or 0 (if the patient needs total care) was attributed for each activity. A total score of 6 represents full autonomy, 4 a moderate impairment of autonomy, and <2 a severe impairment of autonomy (29).

### Baseline Treatment Record

Data on daily treatment were collected from the medical records. Polypharmacy was defined as the prescription of ≥5 medications daily (30). The Drug Burden Index (DBI) was used to assess the sedative and anticholinergic burden of medication (31). The DBI was calculated using the anticholinergic burden calculator developed by the Instituto de BIomedicina de Sevilla (IBIS), available on the Internet (http://www.anticholinergicscales.es/calculate). The DBI is the sum of anticholinergic and sedative effects of every treatment taken by the participant. This effect is calculated using the formula *D*/(δ + *D*), in which *D* is the daily dose taken by the participant and δ the minimum efficacious daily dose approved by the Food and Drug Administration (FDA) and ranges from 0 to 1 for each drug (32). The DBI score is higher if participants take high doses and multiple drugs with sedative and anticholinergic effects.

### Assessment of Baseline Comorbidities

Screening for 49 diseases (listed in **Table 2**) was performed.

The Dementia Screening Questionnaire for Individuals with Intellectual Disabilities (DSQIID) (33) was completed by the referent health worker of the participant to screen for neurocognitive conditions. The first part of this questionnaire targets the participant’s abilities, the second targets the behavior and symptoms usually associated with dementia in people with ID, and the third includes 10 comparative questions. Dementia is suspected for a total score (sum of the second and third parts) of ≥20.

Mental health conditions were assessed using the Reiss Scale (34), designed to screen for mental disorders in people with ID and aged over 12 years. This 38-item scale includes eight sub-scales: aggressive behavior, autism, psychosis, paranoia, depression (behavior symptoms), depression (physiological symptoms), dependence, and avoidance. There are also six maladaptive items, including drug abuse, hyperactivity, self-harm, sexual disorders, suicidal tendencies, and theft. The questionnaire was completed by the referent health worker. Each item is scored from 0 to 2 (0, no problem; 1, problem; 2, severe problem). The presence of co-occurring mental disorders is considered for a score ≥9 for the 26 selected items. The Reiss Scale is used to determine whether the presence of a mental problem for an ID patient has been sufficiently demonstrated. Each sub-scale shows good internal validity (between 0.72 and 0.81), and the French version, developed by Lecavalier and Tassé, shows satisfactory adequacy with the original version of the Reiss Scale (35). A mental disorder was suspected when a current co-occurring psychiatric disease was diagnosed, except ASD and ID. The diagnosis of a mental disorder was established on the basis of a body of evidence: psychiatric symptoms detected by the Reiss Scale, in particular depression and hyperactivity, and the clinical evaluation of investigators (a practitioner and a psychologist).

Other comorbidities were evaluated by examining the medical record (in collaboration with the participant’s general practitioner), the last biological checkup (completed in the year of inclusion), and medical examinations of the participant carried out by one of the authors (SC or SM).

Among the 49 diseases, 44 were grouped into 14 categories of chronic health conditions (detailed in **Table 2**) to provide an overview of the prevalence of the comorbidity categories.

### Determination of the Comorbidity Burden

The revised CIRS-G (36) is the gold standard to evaluate the presence of comorbidities and their medical burden. A total of 14 organ-specific categories are assessed (cardiac, vascular, hematopoietic, respiratory, eye–ear–nose–throat, upper gastrointestinal, lower gastrointestinal, liver, genitourinary system, musculo-skeletal system, neurology, endocrine/metabolic and breast, and psychiatry) (37), with a score between 0 and 4 for each category. 0 indicates no problem, 1 a mild or past significant problem, 2 a moderate problem requiring regular first-line treatment, 3 a severe and chronic problem requiring second-line treatment, and 4 an extremely severe problem requiring acute treatment and involving severe disability. The CIRS-G total score is the sum of each organ-system score.

The severity index (CIRS-SI) is defined as the CIRS-G total score divided by the total number of categories with a score > 1. The participants were separated into two groups according to the CIRS-SI: the *low-severity index group* (CIRS-SI ≤2) and the *high-severity index group* (CIRS-SI >2), as previously described in other studies using these scores (37, 38).

### Statistical Analysis

For analyses of the clinical characteristics associated with the comorbidity burden (CIRS), regression models were used to estimate the association between clinical factors and the CIRS-G score, and CIRS-SI and CIRS-SC components. For analyses of the CIRS-G score, linear regression models were used in which the CIRS-G score was normalized by logarithmic transformation. For the binary components of the CIRS-SC score, logistic regression models were generated. The predictive ability of the models, that is, the concordance rates between the predicted and observed responses, were calculated. The alpha-to-enter was set at 0.2 and alpha-to-exit at 0.10. The significance of adding or removing a variable from the multivariate models was determined by the maximum likelihood ratio test. The goodness of fit of the models was assessed using the Hosmer and Lemeshow test.

First, we described the comorbidities by calculating the prevalence of each in our population. An overview was provided by categorizing the comorbidities into 14 chronic health conditions and calculating the prevalence of each.

Second, we examined the association between comorbidities and clinical characteristics (ASD severity (CARS), level of adaptive functioning (VABS-II scores), level of autonomy (ADL), polypharmacy, and sedative and anticholinergic burden (DBI)). The ADL was analyzed using three sub-groups: low autonomy for a score of 0, 1, or 2; moderate autonomy for 3 or 4; and preserved autonomy for 5 or 6. Analysis of variance (ANOVA), χ^2^, Student–Fisher, or Mann–Whitney tests were applied, depending on the nature of the variables (continuous, dichotomous, or categorized in three levels).

Third, we used multivariate analysis to determine which comorbidity significantly associated with a clinical feature had a dominant effect on this clinical characteristic. The models were adjusted for age. Analysis of covariance (ANCOVA) tests were used for continuous variables (CARS, VABS-II sub-scores, and DBI), polytomous logistic regression for ADL, and logistic regression for dichotomous variables (polypharmacy).

All values are expressed as a percentage or mean ± standard error. The significance level used was 5%. Statistical analyses were performed using SAS version 9.3 (SAS institute, Cary, NC, USA).

## Results

### Patient Characteristics

Overall, 63 adults, with a mean age of 43±15.1 years, were included in our study. The male-to-female ratio was 3.7. Their clinical characteristics are shown in **Table 1**. They had a severe ASD, according to the CARS score (38.9 ± 6.6) and a profound ID, according to the DQ score (57 participants had a DQ score < 20, and 5 had a DQ score between 20 and 30). Gender had no effect on the clinical characteristics (*p* > 0.05).

**Table 1:** Characteristics of the population in the EFAAR study.

		Total sample	Women (*n* = 17)	Men (*n* = 46)	Gender effect (*p* value)
**Age (years)**		42.9 ± 15.1 (21–68)	47.5 ± 14 (23–63)	41.3 ± 15.2 (21–68)	0.21
**ASD severity (CARS)**		38.9 ± 6.6 (25–52)	37.6 ± 7 (25–51.5)	39.4 ± 6.5 (25–52)	0.36
**Adaptive functioning (VABS-II)**	**SS communication**	23.1 ± 7.2 (20–73)	25.4 ± 12.8 (20–73)	22.2 ± 3.2 (21–38)	0.42
	**SS daily life**	23.6 ± 5.7 (20–47)	22.5 ± 2.9 (20–33)	24 ± 6.4 (21–47)	0.92
	**SS social skills**	20.7 ± 2.9 (20–37)	20 ± 0 (20–20)	20.1 ± 3.4 (20–37)	0.22
**Autonomy level (ADL)**		4.2 ± 1.6 (0–6)	3.8 ± 1.6 (0–6)	4.4 ± 1.6 (0–6)	0.13
**Polypharmacy**		58.7%	70.6%	54.3%	0.25
**Sedative and anticholinergic burden (DBI)**		2 ± 1 (0-5.5)	1.9 ± 1 (0–4.1)	2.1 ± 1.1 (0−5.5)	0.73

Values are expressed as percentages or the means ± standard deviation (minimum–maximum).

For the gender effect, the association between gender and every clinical characteristic was assessed using the mean comparison for continuous variables and the χ^2^ test for dichotomous variables.

ADL, activities of daily living; CARS, Childhood Autism Rating Scale; DBI, Drug Burden Index; SS, sub-scores at the VABS-II; VABS-II, Vineland Adaptive Behavior Scale II.

The comorbidity rates are listed in **Table 2**, with the three most frequent being constipation (54%), epilepsy (28.6%), and chronic kidney disease, essentially chronic renal failure (25.4%). Chronic health conditions are also shown in **Table 2**, the most prevalent being gastrointestinal disorders, essentially constipation (55.56%), mental diseases, essentially hyperactivity and depression (39.68%), and neurological diseases, essentially epilepsy (36.51%). In addition, 28.5% of participants had at least one cardiovascular risk factor (hypertension, diabetes, obesity, or dyslipidemia).

**Table 2:** Prevalence of the 14 chronic health conditions and the 49 chronic diseases and their association with clinical characteristics (values depict those without comorbidity vs those with comorbidity).

Chronic health condition	Prevalence (%)	Comorbidity	Prevalence (%)	ASD severity (CARS)	Adaptive functioning (VABS-II)			ADL	Polypharmacy	DBI
					SS communication	SS daily life	SS social skills	Low category (0–2)		
**Hypertension**	13.56	Hypertension	13.56	39.6 ± 6.3 vs 35.9 ± 7	23.1 ± 7.7 vs 23.5 ± 5.5	20.8 ± 3.3 vs 20 ± 0	23.9 ± 6.3 vs 22.9 ± 2	19.6% vs 0%	54.9% vs 75%	2 ± 1.1 vs 1.9 ± 0.8
**Eye disease**	17.46	Glaucoma	0	–	–	–	–	–	–	–
		Blindness and low vision	17.46	39 ± 6.8 vs 38.5 ± 6	23.1 ± 7.7 vs 23 ± 4.7	20.8 ± 3.3 vs 20 ± 0	24 ± 6.2 vs 21.6 ± 0.5	15.4% vs 18.2%	61.5% vs 45.5%	2.1 ± 1.1 vs 1.8 ± 1
**Cardiovascular disease**	15.87	Coronary heart disease	0	–	–	–	–	–	–	–
		Atrial fibrillation	0	–	–	–	–	–	–	–
		Heart failure	7.94	38.9 ± 6.7 vs 38.6 ± 6	23.2 ± 7.5 vs 21.8 ± 0.5	20.8 ± 3.3 vs 20 ± 0	23.7 ± 5.9 vs 22.6 ± 1.3	15.5% vs 20%	60.3% vs 40%	2.1 ± 1.1 vs 1.6 ± 0.8
		Orthostatic hypotension	17.86	37.9 ± 6.2 vs 38.2 ± 8.3	22.8 ± 4.4 vs 21.8 ± 0.5	21.9 ± 4.7 vs 20 ± 0	26.9 ± 8.4 vs 21.8 ± 0.5	4.3% vs 0%	39.1% vs 100%*	1.7 ± 1 vs 2.2 ± 1
		Peripheral vascular disease	4.76	39.3 ± 6.6 vs 31.7 ± 1.5	23.1 ± 7.4 vs 22 ± 0	20.7 ± 3 vs 20 ± 0	23.7 ± 5.8 vs 22 ± 0	16.7% vs 0%	58.3% vs 66.7%	2 ± 1.1 vs 2.3 ± 0.8
**Endocrine disorder**	26.98	Diabetes	3.17	39.1 ± 6.7 vs 34.3 ± 3.2	23.1 ± 7.3 vs 22 ± 0	20.7 ± 3 vs 20 ± 0	23.7 ± 5.8 vs 20 ± 0	16.4% vs 0%	57.4% vs 100%	2 ± 1.1 vs 2.2 ± 0.7
		Thyroid disorders	11.11	39 ± 6.9 vs 38.7 ± 4.5	23 ± 7.4 vs 23.7 ± 5.9	20.8 ± 3.1 vs 20 ± 0	23.9 ± 6 vs 21.6 ± 0.5	16.1% vs 14.3%*	55.4% vs 85.7%	2 ± 1 vs 2.5 ± 1.6
		Obesity	4.76	39.1 ± 6.6 vs 35.8 ± 8.5	22.7 ± 7 vs 30.3 ± 8.3	**20.5 ± 2.6 vs 24.3** ± 7.5*	23.4 ± 5.2 vs 29 ± 13	16.7% vs 0%	58.3% vs 66.7%	2 ± 1.1 vs 2.5 ± 0.7
		Dyslipidemia	12.7	39.3 ± 6.6 vs 36.4 ± 6.6	23 ± 7.6 vs 23.3 ± 4*	20.6 ± 2.7 vs 21.6 ± 4.6	23.6 ± 5.7 vs 25 ± 5.7	16.4% vs 12.5%*	58.2% vs 62.5%	2 ± 1.1 vs 2.3 ± 1
		Other endocrine disease	3.17	38.9 ± 6.6 vs 40 ± 8.5	23.1 ± 7.3 vs 21 ± 0	20.7 ± 3 vs 20 ± 0	23.6 ± 5.7 vs 25 ± 5.7	14.8% vs 50%	59% vs 50%	2 ± 1.1 vs 1.9 ± 0.2
**Joint disease**	15.87	Rheumatoid arthritis. Other inflammatory polyarthropathies and systematic connective tissue disorders	0	–	–	–	–	–	–	–
		Arthrosis	1.59	39 ± 6.6 vs 32.5	23.1 ± 7.3 vs 22	20.7 ± 3 vs 20	23.7 ± 5.7 vs 22	16.1% vs 0%	59.7% vs 0%	2 ± 1.1 vs 1.8
		Osteoporosis with fracture	3.17	38.8 ± 6.5 vs 43.3 ± 11.7	23.1 ± 7.3 vs 21 ± 1.4	20.7 ± 3 vs 20 ± 0	23.7 ± 5.8 vs 21 ± 1.4	14.8% vs 50%	57.4% vs 100%	2 ± 1 vs 2.9 ± 1.7
		Other chronic joint disease	11.11	39.1 ± 6.3 vs 37.5 ± 9.2	23.3 ± 7.6 vs 21.4 ± 0.5	20.8 ± 3.1 vs 20 ± 0	23.9 ± 6 vs 21.4 ± 0.5	16.1% vs 14.3%	57.1% vs 71.4%	2 ± 1.1 vs 1.8 ± 1
**Lung disease**	7.94	Chronic obstructive pulmonary disease	3.17	38.9 ± 6.6 vs 39.5 ± 9.9	22.9 ± 7.1 vs 29.5 ± 10.6	20.7 ± 3 vs 20 ± 0	23.7 ± 5.8 vs 22 ± 0	16.4% vs 0%	57.4% vs 100%	2 ± 1.1 vs 1.6 ± 0.3
		Asthma	4.76	38.9 ± 6.7 vs 38.8 ± 6.7	23.2 ± 7.4 vs 21 ± 0	20.7 ± 3 vs 20 ± 0	23.5 ± 5.8 vs 25.7 ± 4.2	16.7% vs 0%	58.3% vs 66.7%	2 ± 1 vs 2.2 ± 1.5
		Bronchiectasis	0	–	–	–	–	–	–	–
**Gastrointestinal disease**	55.56	Inflammatory bowel disease	0	–	–	–	–	–	–	–
		Diverticular disease of intestine	1.59	39.1 ± 6.6 vs 30	23.1 ± 7.3 vs 22	20.7 ± 3 vs 20	23.7 ± 5.7 vs 22	16.1% vs 0%	58.1% vs 100%	2 ± 1.1 vs 2.6
		Dyspepsia	11.11	39.3 ± 6.4 vs 36.5 ± 7.9	23.1 ± 7.7 vs 22.9 ± 3.8	20.6 ± 2.7 vs 21.4 ± 4.3	23.5 ± 5.4 vs 24.1 ± 7.5	14.8% vs 22.2%	53.7% vs 88.9%	2 ± 1.1 vs 2.2 ± 0.7
		Irritable bowel syndrome	0	–	–	–	–	–	–	–
		Constipation	53.97	38.1 ± 6.7 vs 39.6 ± 6.6	23.2 ± 4.7 vs 22.9 ± 8.9	21.5 ± 4.2 vs 20 ± 0*	26 ± 7.7 vs 21.6 ± 0.8**	13.8% vs 17.6%*	27.6% vs 85.3%***	1.6 ± 0.8 vs 2.3 ± 1.1**
**Mental disease**	39.68	Depression	11.11	38.8 ± 6.5 vs 39.4 ± 8	22.1 ± 2.9 vs 30.7 ± 19.6*	20.8 ± 3.1 vs 20 ± 0	23.6 ± 5.9 vs 23.6 ± 4.4	16.1% vs 14.3%	57.1% vs 71.4%	2 ± 1 vs 2.1 ± 1.4
		Anxiety and other neurotic stress-related and somatoform disorders	3.17	39 ± 6.7 vs 36.5 ± 5	23.1 ± 7.3 vs 21.5 ± 0.7	20.7 ± 3 vs 20 ± 0	23.7 ± 5.8 vs 21.5 ± 0.7	16.4% vs 0%	57.4% vs 100%	2 ± 1 vs 2.6 ± 1.2
		Alcohol problems	0	–	–	–	–	–	–	–
		Other psychoactive substance misuse	0	–	–	–	–	–	–	–
		Schizophrenia. Related non-organic psychosis	3.17	39.2 ± 6.5 vs 30.3 ± 6.7	22.3 ± 3.4 vs 47.5 ± 36.1	20.7 ± 3 vs 20 ± 0	23.7 ± 5.8 vs 22 ± 0	16.4% vs 0%	57.4% vs 100%	2 ± 1.1 vs 1.6 ± 0.3
		Hyperactivity	22.22	38.2 ± 6.7 vs 41.4 ± 5.7	22.5 ± 3.7 vs 25 ± 13.8	20.9 ± 3.3 vs 20 ± 0	24 ± 6.3 vs 22.2 ± 2.2	10.2% vs 35.7%	51% vs 85.7%*	1.9 ± 1 vs 2.4 ± 1.1
		Anorexia or bulimia	9.52	39.1 ± 6.5 vs 37 ± 7.8	23.1 ± 7.5 vs 22.8 ± 3.1	20.6 ± 2.8 vs 21.7 ± 4.1	23.4 ± 5.1 vs 25.8 ± 10.4	15.8% vs 16.7%	59.6% vs 50%	2 ± 1.1 vs 1.9 ± 0.5
**Stroke**	3.17	Stroke and transient ischemic attack	3.17	38.9 ± 6.7 vs 38.5 ± 2.8	23.1 ± 7.3 vs 22 ± 0	20.7 ± 3 vs 20 ± 0	23.7 ± 5.8 vs 22 ± 0	16.4% vs 0%	57.4% vs 100%	2 ± 1.1 vs 2.2 ± 0.6
**Cancer**	3.17	Cancer in last 5 years	3.17	38.9 ± 6.7 vs 38.3 ± 4.6	22.3 ± 3.4 vs 47.5 ± 36.1	20.7 ± 3 vs 20 ± 0	23.7 ± 5.8 vs 22	16.4% vs 0%	57.4% vs 100%	2 ± 1.1 vs 2.1 ± 0.4
**Kidney disease**	25.39	Chronic kidney disease	25.39	40.6 ± 6.4 vs 35.2 ± 6.9**	22.1 ± 3.1 vs 26.7 ± 13.2**	20.8 ± 3.3 vs 20.8 ± 3.3	24 ± 6.3 vs 23.3 ± 5.5	22.2% vs 6.3%	58.3% vs 68.8%	2 ± 1.2 vs 2.1 ± 0.8
**Neurological disease**	36.51	Parkinson’s disease	7.94	39.1 ± 6.7 vs 36.6 ± 5.2	23.2 ± 7.5 vs 21.6 ± 0.6	20.7 ± 3.1 vs 20 ± 0	23.7 ± 5.9 vs 22.4 ± 1.5	17.2% vs 0%	58.6% vs 60%	2 ± 1.1 vs 2.4 ± 1
		Epilepsy	28.57	37.2 ± 6.3 vs 43.3 ± 5.3***	23.4 ± 8.2 vs 22.2 ± 4	20.6 ± 2.4 vs 20.9 ± 4	24 ± 5.9 vs 22.7 ± 5.2	11.1% vs 27.8%	57.8% vs 61.1%	2 ± 1.1 vs 2.1 ± 0.9
		Dementia	3.17	39.2 ± 6.4 vs 30 ± 7.1	22.3 ± 3.4 vs 47.5 ± 36.1	20.7 ± 3 vs 20 ± 0	23.7 ± 5.8 vs 22 ± 0	16.4% vs 0%	57.4% vs 100%	2 ± 1.1 vs 2.4 ± 0.8
		Migraine	3.17	38.9 ± 6.6 vs 38 ± 8.5	23.1 ± 7.3 vs 21.5 ± 0.7	20.7 ± 3 vs 20 ± 0	23.7 ± 5.8 vs 21.5 ± 0.7	16.4% vs 0%	59% vs 50%	2 ± 1.1 vs 1.9 ± 0.3
		Multiple sclerosis	0	–	–	–	–	–	–	–
**Liver disease**	7.94	Viral hepatitis	0	–	–	–	–	–	–	–
		Chronic liver disease	7.94	39.4 ± 6.1 vs 33.4 ± 10.4*	22.9 ± 7.3 vs 25.2 ± 5.5	20.3 ± 2.3 vs 24.6 ± 6.4**	3 ± 4.2 vs 31 ± 13.3	15.5% vs 20%	56.9% vs 80%	2 ± 1 vs 2.5 ± 1.1
**Immune dysfunction**	23.81	Allergy	9.84	38.6 ± 6.6 vs 41.1 ± 6.5	22.4 ± 3.6 vs 27 ± 17.3	20.8 ± 3.2 vs 20 ± 0	23.9 ± 6.1 vs 22 ± 1.9	16.7% vs 11.1%	57.4% vs 66.7%	2 ± 1.1 vs 1.6 ± 1
		Psoriasis or eczema	1.59	38.7 ± 6.8 vs 40.9 ± 4.2	22 ± 2.8 vs 33.2 ± 20.5**	20.5 ± 2.2 vs 22.8 ± 6.9	23.4 ± 5.4 vs 25.5 ± 8.6	14% vs 33.3%	61.4% vs 33.3%	2 ± 1.1 vs 2 ± 0.5
**Others**		Undernutrition	4.76	38.6 ± 6.7 vs 44.3 ± 1.8	23.2 ± 7.4 vs 21.3 ± 0.6	20.7 ± 3 vs 20 ± 0	23.7 ± 5.8 vs 21.3 ± 0.6	16.7% vs 0%	56.7% vs 100%	2.1 ± 1 vs 0.9 ± 0.8*
		Hearing loss	9.52	39 ± 6.6 vs 33	23.1 ± 7.3 vs 22	20.7 ± 3 vs 20	23.7 ± 5.7 vs 22	16.1% vs 0%	58.1% vs 100%	2 ± 1 vs 3.2
		Chronic anemia	17.54	38.4 ± 6.9 vs 42.1 ± 5.44	23.7 ± 8.3 vs 21.2 ± 0.6*	20.9 ± 3.4 vs 20 ± 0	24 ± 6.3 vs 21.6 ± 1.4	19.1% vs 10%	57.4% vs 90%	2 ± 1 vs 2.1 ± 1.3
		Painful condition	4.76	38.9 ± 5.8 vs 43.6 ± 8.8	23.3 ± 7.7 vs 21.3 ± 0.8	20.8 ± 3.1 vs 20 ± 0	23.9 ± 6 vs 21.3 ± 0.8	14.5% vs 33.3%	56.4% vs 66.7%	1.9 ± 1.1 vs 2.7 ± 0.9
		Prostate disorders	14.29	38.8 ± 6.7 vs 40.5 ± 4.6	23.2 ± 7.4 vs 21.3 ± 0.6	20.7 ± 3 vs 20 ± 0	23.7 ± 5.8 vs 21.3 ± 0.6	16.7% vs 0%	58.3% vs 66.7%	2 ± 1.1 vs 2.2 ± 0.9

Among 49 chronic diseases, 44 are placed into 14 chronic health conditions detailed in the first column. Prevalence is expressed as a percentage.

The results for continuous variables for the group without comorbidities versus that with are expressed as the means ± standard error.

The results for dichotomous variables for the group without comorbidities versus that with are expressed as percentages. For polypharmacy, results are expressed in percentage for the group without versus with the comorbidity. For example, 54.9% of patient without hypertension have polypharmacy, whereas 75% of patients with hypertension have polypharmacy. For ADL category, results are expressed in percentage of patients without versus with the comorbidity only for the “low autonomy” category (ADL score between 0 and 2). For example, 19.6% of patients without hypertension have a low score at ADL, whereas 0% of patients with hypertension have a low score at ADL.

ANOVA, χ^2^, Student–Fisher, or Mann–Whitney tests were applied, depending on the nature of the variables (continuous, dichotomous, or categorized in three levels). p values are expressed as ranges. No symbol: nonsignificant (p value > 0.05), *0.05 ≤ p < 0.01, **0.01 ≤ p < 0.001, ***p ≤ 0.001.

ADL, activities of daily living; CARS, Childhood Autism Rating Scale; DBI, Drug Burden Index; SS, sub-scores at the VABS-II; VABS-II, Vineland Adaptive Behavior Scale II.

### Association Between Clinical Characteristics and Comorbidities in ASD-ID Patients

We examined the extent to which the seven clinical characteristics of ASD-ID patients [ASD severity (CARS), adaptive functioning (VABS-II sub-scores), autonomy (ADL), polypharmacy, and sedative and anticholinergic burden (DBI)] are associated with each comorbidity and performed multivariate analyses adjusted for age to determine the weight of such comorbidity on these seven clinical characteristics (**Table 2**).

A more severe ASD was associated with epilepsy, whereas lower ASD severity was associated with chronic kidney disease and chronic liver disease, as well as cardiovascular risk factors (*p* value of 0.03). Multivariate analysis showed that only epilepsy was correlated with ASD severity (*p* value of 0.0128, adjusted *R* ^2^ of 0.217; having epilepsy increased the CARS score by 5.13).

Lower VABS-II communication sub-scores were associated with chronic kidney disease, dyslipidemia, and chronic anemia. Higher scores were associated with psoriasis and eczema. After multivariate analysis, psoriasis and eczema still were correlated with the VABS-II communication sub-score (*p* value of 0.0036, adjusted *R* ^2^ of 0.189; having psoriasis or eczema increased the communication sub-score by 9.8). Constipation was the only comorbidity associated with lower VABS-II social skills sub-scores (*p* value of 0.001). Lower VABS-II daily-life sub-scores were associated with constipation, whereas higher scores were associated with obesity and chronic liver disease. After multivariate analyses, chronic liver disease and age still were correlated with daily-life sub-scores (respectively *p* = 0.02 and 0.004, respectively, adjusted *R* ^2^ of 0.256; having chronic liver disease increased the daily-life sub-score by 4.4, and being older decreased the daily-life sub-score by 0.05).

The ADL score was associated with thyroid disorders, dyslipidemia, and constipation (see **Supplementary Table 1** for details). No associations remained after multivariate analyses.

Higher polypharmacy was associated with orthostatic hypotension, constipation, and hyperactivity symptoms. Logistic regression showed that polypharmacy is associated with an 11.8-fold increased risk of constipation (OR = 11.8; 95% CI 3.25–42.97). Similarly, a high DBI was associated with constipation, whereas a low DBI was associated with undernutrition. The variable “undernutrition” could not be entered into the multivariate model because only three patients showed undernutrition.

### Determination of Comorbidity Burden

The mean CIRS-G total score was 10.6±4.8. In univariate analyses, the log(CIRS-G total score) was significantly associated with age (p < 0.0001), low Vineland II daily-life and social-skills sub-scores (*p* = 0.03 and 0.01, respectively), a low level of autonomy assessed by the ADL (*p* < 0.001), polypharmacy (*p* = 0.0001), and a sedative and anticholinergic burden assessed by the DBI (*p* = 0.005). The results of multivariate analyses for the log(CIRS-G total score) are shown in **Table 3**. The CIRS-G total score was significantly predicted by age (*p* = 0.001), polypharmacy (*p* < 0.0001), and a low level of autonomy assessed by the ADL (*p* < 0.0001) (*R* ^2^ of 0.55, *p* < 0.0001). Univariate and multivariate analyses performed for the CIRS-G total score without logarithmic transformation gave similar results. Furthermore, inflammation (defined as a C-reactive protein concentration >5 mg/mL) was significantly associated with the log(CIRS-G total score) (*p* = 0.004), but not age (*p* = 0.17).

**Table 3:** ANCOVA analysis of CIRS total scores (log CIRS tot) by covariable [selected forward with the best Akaike information criterion (AIC)].

	log CIRS-G		
	*Beta (SE)*	*[95% CI]*	*p value*
**Intercept**	2.1 (0.2)	[1.3; 2.7]	<0.0001
**Age**	0.009 (0.003)	[0.003; 0.01]	0.001
**SS daily life**	–	–	–
**SS social skills**	–	–	–
**ADL**	−0.1 (0.02)	[−0.1; −0.04]	<0.0001
**Polypharmacy (1/0)**	0.4 (0.08)	[0.1; 0.5]	<0.0001
**DBI**	–	–	–
**R² total**	0.55		

Linear regressions were used when the CIRS-G score was normalized by logarithmic transformation because of its distribution. Dashes indicate that the variable was not entered into the model.

SE, standard error; CI, confidence interval; ADL, activities of daily living; DBI, Drug Burden Index; SS, sub-score at VABS-II (Vineland Adaptive Behavior Scale II).

The mean CIRS-SI was 2.46 ± 0.5, with 73% of participants in the *high-severity index group*. Univariate analyses showed no significant associated factors for the CIRS-SI.

The mean CIRS-SC score was 1.79 ± 1.03, with 49% of participants in the *high-severity comorbidity group*. Univariate analyses showed that the *high-severity comorbidity group* was older (*p* = 0.005) and had lower Vineland II daily-life and social-skills sub-scores (*p* = 0.02 and 0.04, respectively), a lower level of autonomy assessed by the ADL (*p* = 0.02), more frequent polypharmacy (*p* = 0.003), and a higher sedative and anticholinergic burden assessed by the DBI (*p* = 0.001). The results of logistic regressions are shown in **Table 4**. They showed that the older the participants and the higher their DBI, the higher the CIRS-SC score (OR = 1.1, *p* = 0.0025, and OR = 3.1, *p* = 0.002, respectively).

**Table 4:** Logistic regression analysis of factors related to CIRS-SC categories.

Risk factors	Unit	ORa*	95% CI	*p* value
**Age**	5	1.1	(1.1; 1.7)	0.0025
**DBI**	1	3.1	(1.4; 6.6)	0.002

*Adjusted odds ratio; concordance rate: 83.5%; Hosmer and Lemeshow test = 0.17.

The UNITS statement makes it possible to specify the units of change for continuous explanatory variables so that customized odds ratios can be estimated.

DBI, Drug Burden Index.

Given the high and unexpected prevalence of chronic kidney diseases, we explored the possible causes of such kidney impairment. There was a positive correlation between age and cardiovascular risk factors (*p* = 0.02), age and chronic kidney disease (*p* = 0.005), and cardiovascular risk factors and chronic kidney disease (*p* = 0.0009).

## Discussion

We provide a detailed qualitative and quantitative description of comorbidities in a well-phenotyped cohort of adult patients with ASD and ID. Our analyses provide new information concerning the weight of such comorbidities by showing that the comorbidity burden is associated with age, autonomy, polypharmacy, and sedative and anticholinergic burden. Our study is the first to explore the comorbidity burden in ageing ASD-ID patients using the CIRS-G. The distribution of comorbidities shows the extent to which they are common in ASD-ID patients during adulthood and the pre-elderly period. Analyses of the associations between such comorbidities and the clinical characteristics of ASD could indicate future directions to promote personalized medicine for ageing ASD patients.

### Potential Shortcomings and Limitations of the Interpretations

The EFAAR study is the first with a multicentric and prospective design carried out on adult patients with ASD-ID in France. With only 63 patients, our ASD-ID cohort may not be representative of all people with ASD-ID in France. In addition, our patients were recruited from medico-social institutions. They were not hospitalized at the time of the assessment and were considered to be stable. Nevertheless, we may have selected individuals with more severe ASD-ID, as shown by the mean DQ. Thus, the high comorbidity burden and rates found in our study should be interpreted with caution, because it refers to a very specific population with a very severe ASD-ID disorder. The severe ID observed in our population could be the most important cause of the observed high comorbidity burden.

The homogeneity of the profound ID prevented us from using the level of ID as a variable in univariate and multivariate analyses. Thus, the results of this preliminary study need to be confirmed in a larger cohort of ageing people with ASD, with or without ID, to better understand the effect of ID on the comorbidity burden.

The colinearity of the clinical characteristics and certain comorbidities also make interpretation of the univariate analyses difficult.

Furthermore, there are no previous studies concerning ageing with ASD-ID. Thus, we can compare our results only with those obtained for ageing people with ID.

Mental disorders were diagnosed on the basis of a screening scale (Reiss Scale) and clinical evaluation. Although there are no standardized tools to diagnose mental disorders, such as depression or hyperactivity, in the ASD-ID population, underdiagnosis or overdiagnosis of mental disorders could have been made, introducing a measurement bias.

### Integration of the Discovery Into Current Understanding of the Problem

The comorbidity burden, assessed by the CIRS-G total score, of our ASD-ID population, with a mean age of 42.9 years, was comparable with that of an older population (with a mean age of 79 years) from the general hospitalized population in a geriatric department (37). The CIRS-SI of our sample was also higher than that of a population with a mean age of approximately 80 years, supporting the hypothesis of premature ageing in ASD-ID, partially due to a high comorbidity burden. Furthermore, elderly people from the general population often show chronic and low-level inflammation, due to an imbalance between proinflammatory and anti-inflammatory cytokines, called inflamm-ageing, which is associated with multimorbidity and frailty (40). A specific serum inflammation profile has been observed in ASD (41), and we observed a significant association between the CIRS-G score and elevated CRP levels in our ASD-ID cohort (data not shown). This inflamm-ageing process could thus partially explain such a comorbidity burden and be an indirect cause of pathological and/or premature ageing in ASD. However, more precise tools for assessing inflammation and, in particular, microinflammation, such as the measurement of serum orosomucoid or interleukin 6 (IL-6) serum levels, would be useful to further explore this hypothesis.

In multivariate analyses, the comorbidity burden (assessed by the CIRS-G total score) correlated with higher age, lower autonomy, and higher polypharmacy. The level of autonomy assessed by the ADL is significantly associated with higher age in the general population (42). Polypharmacy is associated with multimorbidity in the general population (3) and can increase the risk of decreased autonomy in the geriatric population (43). Thus, these three factors (age, autonomy, and polypharmacy) could synergize to increase the comorbidity burden in ageing ASD-ID people. Focusing on promoting autonomy and reducing polypharmacy in older ASD-ID patients could reduce their comorbidity burden and thus reduce the impact of pathological ageing. Comprehensive geriatric assessment (CGA) is a multidimensional and multidisciplinary process used to identify the needs of patients to reduce morbidity and mortality and promote their autonomy (44). Given the factors associated with the comorbidity burden in our study, CGA could be an interesting basis from which to propose the medical management of ageing ASD-ID patients. In light of the associations observed between these three clinical characteristics (age, autonomy, and polypharmacy) and certain comorbidities in our study, courses of action could be proposed for daily clinical practice to reduce the comorbidity burden. Autonomy was not associated with any specific comorbidity in multivariate analyses. Thus, its management must be more global than a targeted action on one associated disease of ASD-ID patients. Multivariate analyses revealed an association between polypharmacy and constipation. Thus, special attention towards treating constipation in connection with reducing polypharmacy could have a positive impact on the comorbidity burden.

In the general population, the CIRS-SC score correlates with the multimorbidity prognosis (45) and reflects the number of comorbidity categories with a severe degree of illness. Multivariate analyses showed the CIRS-SC score to positively correlate with age and sedative and anticholinergic burden assessed by the DBI and the DBI to be associated with polypharmacy. An increase of the DBI by 1 point increased the CIRS-SC score by 3.1 points, showing the important weight of the sedative and anticholinergic burden in the severity of comorbidity, probably due to higher polypharmacy. Furthermore, our ASD-ID population had a higher DBI score (2 ± 1.1) than those of an ID population aged over 50 years (1.1 ± 1.73) (18) and general population patients hospitalized in a medical service with a mean of age of 85 years (between 0.53 and 0.64) (46). Thus, the higher DBI score we observed could be due to the severe ID of our population, the co-occurrence of ASD, or simply the resulting high comorbidity burden. A high DBI was associated with constipation, probably because of the side effects of the psychotropic medications in our sample, which needs to be more precisely evaluated. The DBI could thus be a useful tool to improve pharmacological treatment in ASD-ID, all the more since the misuse of psychotropic drugs has been demonstrated for approximately one third of ASD patients due to the lack of a consensus on pharmacological treatment for ASD (47).

The three most common chronic health conditions in our ASD-ID population, with an average age of 43 years, were gastrointestinal (56%), mental (40%), and neurological disorders (37%). There are no data concerning the frequency of these chronic health conditions in ASD patients with ID. The reported prevalence of these chronic health conditions is heterogeneous, depending on whether the ASD or ID population was considered.

The general reported prevalence of gastrointestinal disorders varies between 30% and almost 90% in ASD (14, 15) and has been estimated to be 17% in ID patients (48), suggesting that gastrointestinal disorders are a comorbid condition of ASD, rather than ID (49).

The reported prevalence of mental disorders in ASD children varies between 26% and 70% (50), is approximately 34% in young adults (51), and reaches 54% in ASD adults with an average age of 39 years (17), whereas 16.6% to 48% of ID adults have mental disorders (52–54) and only 9.2% of those of the general population (51). Thus, the prevalence of mental disorders appears to be comparable between the ASD and ID population, and the rate observed in our study is concordant with that of the literature. Studies exploring mental comorbidities in ASD adults of approximately 40 years of age have reported depression rates between 10% and 69% (55–58), similar to the prevalence found in our study. A recent meta-analysis concluded that the prevalence of current depression in ASD adults is 23% (59), whereas a prevalence of 14.7% to 39% has been reported for an ID population aged over 50 years (48). The rate of 11% observed in our study appears to be low relative to the prevalence of depression previously reported for ASD and ID. A recent study in young adults showed depression in 24.1% of ASD patients without ID, 9.1% in ASD patients with ID, and 6% in patients without ASD or ID (60). The authors emphasized the difficulty of diagnosing depression in ASD-ID patients to explain the reduced prevalence of depression when ASD was associated with ID. It is possible that depression was also underdiagnosed in our study because of the difficulty for patients with severe ASD and ID to verbalize their symptoms. The moderate significant association between depression and high VABS-II communication sub-scores in our study reinforces this argument, leading us to believe that we can detect depression only in mild or moderate ID patients. Depression is also influenced by the level of ID and was shown to be 10% lower in the ASD-ID population in the recent meta-analysis conducted by Hollocks et al. (59), and our population showed profound ID, reflected by the very low DQ scores. Here, we used a standardized tool to detect psychiatric comorbidities, in particular depression, for which two aspects were screened by the Reiss Scale: behavioral and physiological depressive symptoms. Although this scale is only a screening tool, the use of behavioral and physiological indicators appears to be well adapted for ASD-ID patients. Nevertheless, the complexity of diagnosing mental disorders in ASD-ID patients highlights the necessity to develop specific scales to detect these overlapping diseases (61).

Neurological disorders were the third most common chronic health condition in our ASD-ID population. Epilepsy was found in 29% of participants. This disorder has a general prevalence of between 11% and 39% in ASD (62), with no increase with age (63), whereas it occurs in 24.1% of the ID population aged over 50 years (48), compared with only 1% in the general population (64). These data suggest comparable epilepsy rates in ASD and ID, without any additive effect of ASD and ID in our cohort.

In conclusion, the heterogeneity of the assessment methods used can at least partially explain the large range of the prevalence of these three chronic health conditions in ASD reported in the literature (59).

Our study highlights a surprisingly high rate of chronic kidney disease (25%) in ASD-ID patients, whereas only 15% of ID patients with an average age of 62 years have been reported to have this condition (65). We thus explored the possible causes. We observed a positive correlation between age and cardiovascular risk factors, age and chronic kidney disease, and cardiovascular risk factors and chronic kidney disease. Thus, chronic kidney disease was associated with age, probably due to a higher frequency of cardiovascular risk factors in older participants, which is commonplace in the general population (66). Chronic kidney disease was also more common in women in our sample (data not shown), without any physiological explanation.

In multivariate analyses, ASD severity positively correlated with epilepsy, as already described in literature (67). The IQ level appears to be the most dominant risk factor of epilepsy in the ASD population, more than ASD severity (68). However, the DQ of our cohort showed a profound and homogeneous ID in our population, which prevented us from evaluating the association between ID level and epilepsy. A high VABS-II communication sub-score positively correlated with psoriasis and eczema in multivariate analysis. This association could be explained by the underdiagnosis of dermatological affections in more severe ASD-ID patients, who cannot notify the general practitioner of their symptoms or for whom a complete clinical examination can be more difficult. In multivariate analyses, a lower VABS-II social skills sub-score was associated with constipation, which is consistent with the common observation of an association between ASD severity and gastrointestinal disorders (49, 69). A higher VABS-II daily-life sub-score was associated with chronic liver diseases in multivariate analyses, without any explanation. This association needs to be tested in a larger cohort to develop a pathophysiological hypothesis. Finally, polypharmacy and a high DBI were associated with constipation in multivariate analyses, likely due to the over-prescription of psychotropic drugs. These associations could be used for the promotion of personalized medical care of ASD-ID patients to assess their comorbidities according to clinical features in daily practice.

### Future Directions

The ageing of people with ASD-ID could have an additive effect on their comorbidity burden and its prevalence, likely resulting in pathological ageing. Our results highlight the necessity of assessing gastrointestinal, mental, and neurological disorders, as well as chronic kidney disease and cardiovascular risk factors in ageing ASD-ID patients. Comorbidities need to be evaluated to reduce conflicting treatment and prevent polypharmacy and its iatrogenic effects. The use of the CIRS-G in clinical practice could help practitioners to reduce the comorbidity burden and promote autonomy. The research of specific comorbidities, such as epilepsy, cutaneous diseases, and constipation, based on the clinical characteristics of the ASD-ID patient, should be generalized.

Polypharmacy, multimorbidity and its associated problems, and frailty, three major geriatric concerns, must be investigated to propose personalized geriatric medical care for ASD-ID patients.

Because our population had profound ID, we also need to investigate geriatric syndromes in a large cohort of ASD patients, with and without ID, to evaluate the influence of ID on the comorbidity burden, as well as the prevalence of geriatric syndromes. Data sharing with a general population cohort of adults and pre-elderly people, such as that of CONSTANCES, could also help us to compare the prevalence of comorbidities and reinforce the hypothesis of premature ageing in the ASD-ID population.

## Ethics Statement

Authorization for handling personal data was granted by the French Data Protection Authority (CNIL: Commission Nationale de l’Informatique et Libertés). The initial project was approved by the French Ethical Research Committee (Comité de Protection de Personnes (CPP), identification number 2016-A00166-45) and registered in the international clinical trials register (number NCT02791321). All subjects or their legal representative gave written informed consent in accordance with the Declaration of Helsinki.

## Author Contributions

SM, TA, and AB drafted the manuscript. SM, CM, and AB revised the manuscript. SCo, SCr, and SM collected the data. CM and SM conducted the statistical analyses. M-CP, AB, SM, ÉP, VG, CJ, and HB designed the EFAAR study. JL and CM monitored the data of the EFAAR study. AB and SM coordinated the EFAAR study. AB is the principal investigator of the EFAAR study.

## Funding

The EFAAR study is financially supported by a CNSA-IResP grant. TNA has been supported by the Center of Excellence in Neurodegenerative Diseases (CoEN) of Montpellier.

## Conflict of Interest Statement

The authors declare that the research was conducted in the absence of any commercial or financial relationships that could be construed as a potential conflict of interest.
