# Supplementary material for: Comorbidity Burden in Adults With Autism Spectrum Disorders and Intellectual Disabilities—A Report From the EFAAR (Frailty Assessment in Ageing Adults With Autism Spectrum and Intellectual Disabilities) Study
Source: Front Psychiatry. 2019 Sep 19;10:617. doi: 10.3389/fpsyt.2019.00617 (PMC6761800; doi:10.3389/fpsyt.2019.00617)
Supplement: Supplementary file 1 [file Table_1.docx]

**Supplementary table 1:**

Association between the 49 comorbidities and each ADL category (Values depict those without comorbidity vs. those with comorbidity).

| **comorbidity** | **ADL score category** | | |
| --- | --- | --- | --- |
|  | **0-2** | **3-4** | **5-6** |
| hypertension | 19.6% vs 0% | 31.4% vs 12.5% | 49% vs 87.5% |
| glaucoma | - | - | - |
| blindness and low vision | 15.4% vs 18.2% | 36.5% vs 9.1% | 48.1% vs 72.7% |
| coronary heart disease | - | - | - |
| atrial fibrillation | - | - | - |
| heart failure | 15.5% vs 20% | 34.5% vs 0% | 50% vs 80% |
| orthostatic hypotension | 4.3% vs 0% | 26.1% vs 60% | 69.6% vs 40% |
| peripheral vascular disease | 16.7% vs 0% | 31.7% vs 33.3% | 51.7% vs 66.7% |
| diabetes | 16.4% vs 0% | 29.5% vs 100% | 54.1% vs 0% |
| thyroid disorders | **16.1% vs 14.3%*** | **26.8% vs 71.4%*** | **57.1% vs 14.3%*** |
| obesity | 16.7% vs 0% | 33.3% vs 0% | 50% vs 100% |
| dyslipidemia | **16.4% vs 12.5%*** | **25.5% vs 75%*** | **58.2% vs 12.5%*** |
| other endocrine disease | 14.8% vs 50% | 32.8% vs 0% | 52.5% vs 50% |
| rheumatoid arthritis. other inflammatory polyarthropathies and systematic connective tissue disorders | - | - | - |
| arthrosis | 16.1% vs 0% | 30.6% vs 100% | 53.2% vs 0% |
| osteoporose with fracture | 14.8% vs 50% | 31.1% vs 50% | 54.1% vs 0% |
| other chronic joint disease | 16.1% vs 14.3% | 30.4% vs 42.9% | 53.6% vs 42.9% |
| chronic obstructive pulmonary disease | 16.4% vs 0% | 31.1% vs 50% | 52.5% vs 50% |
| asthma | 16.7% vs 0% | 31.7% vs 33.3% | 51.7% vs 66.7% |
| bronchiectasis | - | - | - |
| inflammatory bowel disease | - | - | - |
| diverticular disease of intestine | 16.1% vs 0% | 30.6% vs 100% | 53.2% vs 0% |
| dyspepsia | 14.8% vs 22.2% | 29.6% vs 44.4% | 55.6% vs 33.3% |
| irritable bowel syndrome | - | - | - |
| constipation | **13.8% vs 17.6%*** | **17.2% vs 44.1%*** | **69% vs 38.2%*** |
| depression | 16.1% vs 14.3% | 33.9% vs 14.3% | 50% vs 71.4% |
| anxiety and other neurotic. stress related and somatoform disorders | 16.4% vs 0% | 32.8% vs 0% | 50.8% vs 100% |
| alcohol problems | - | - | - |
| other psychoactive substance misuse | - | - | - |
| schizophrenia. related non-organic psychosis. | 16.4% vs 0% | 32.8% vs 0% | 50.8% vs 100% |
| hyperactivity | 10.2% vs 35.7% | 34.7% vs 21.4% | 55.1% vs 42.9% |
| anorexia or bulimia | 15.8% vs 16.7% | 29.8% vs 50% | 54.4% vs 33.3% |
| strocke and transient ischaemic attack | 16.4% vs 0% | 29.5% vs 100% | 54.1% vs 0% |
| cancer in last five years | 16.4% vs 0% | 31.1% vs 50% | 52.5% vs 50% |
| chronic kidney disease | 22.2% vs 6.3% | 25% vs 56.3% | 52.8% vs 37.5% |
| parkinson's disease | 17.2% vs 0% | 31% vs 40% | 51.7% vs 60% |
| epilepsy | 11.1% vs 27.8% | 31.1% vs 33.3% | 57.8% vs 38.9% |
| dementia | 16.4% vs 0% | 31.1% vs 50% | 52.5% vs 50% |
| migraine | 16.4% vs 0% | 31.1% vs 50% | 52.5% vs 50% |
| multiple sclerosis | - | - | - |
| viral hepatitis | - | - | - |
| chronic liver disease | 15.5% vs 20% | 32.8% vs 20% | 51.7% vs 60% |
| allergy | 16.7% vs 11.1% | 31.5% vs 33.3% | 51.9% vs 55.6% |
| psoriasis or eczema | 14% vs 33.3% | 33.3% vs 16.7% | 52.6% vs 50% |
| undernutrition | 16.7% vs 0% | 31.7% vs 33.3% | 51.7% vs 66.7% |
| hearing loss | 16.1% vs 0% | 32.3% vs 0% | 51.6% vs 100% |
| chronic anemia | 19.1% vs 10% | 34% vs 40% | 46.8% vs 50% |
| painful condition | 14.5% vs 33.3% | 29.1% vs 50% | 56.4% vs 16.7% |
| prostate disorders | 16.7% vs 0% | 30% vs 66.7% | 53.3% vs 33.3% |

The ADL category 0-2 corresponds to a low autonomy, 3-4 to a moderate autonomy impairment, and 5-6 to a conserved autonomy.

Results are expressed in percentage of patients without *versus* with the comorbidity per category. For example, 19.6% of patients without hypertension have a low score at ADL, 31.4% of them have an intermediate score at ADL and 49% of them have a high score at ADL, when 0% of patients with hypertension have a low score at ADL, 12.5% of them have an intermediate score and 87.5% of them have a high score at ADL.

P-values are expressed as ranges. No symbol: non significant (p value>0.05), *0.05 ≤ p <0.01, *0.01 ≤ p < 0.001, *** p ≤0.001.

ADL: Activities in Daily Life
